# Evidence for and against Liquid-Liquid Phase Separation in the Nucleus

**DOI:** 10.3390/ncrna5040050

**Published:** 2019-11-01

**Authors:** Peng A, Stephanie C. Weber

**Affiliations:** 1 Department of Biology, McGill University, Montreal, QC H3A 1B1, Canada; peng.a@mail.mcgill.ca; 2 Department of Physics, McGill University, Montreal, QC H3A 2T8, Canada

**Keywords:** liquid-liquid phase separation, nuclear bodies, nucleolus, heterochromatin, paraspeckles, transcriptional condensates, replication compartments

## Abstract

Enclosed by two membranes, the nucleus itself is comprised of various membraneless compartments, including nuclear bodies and chromatin domains. These compartments play an important though still poorly understood role in gene regulation. Significant progress has been made in characterizing the dynamic behavior of nuclear compartments and liquid-liquid phase separation (LLPS) has emerged as a prominent mechanism governing their assembly. However, recent work reveals that certain nuclear structures violate key predictions of LLPS, suggesting that alternative mechanisms likely contribute to nuclear organization. Here, we review the evidence for and against LLPS for several nuclear compartments and discuss experimental strategies to identify the mechanism(s) underlying their assembly. We propose that LLPS, together with multiple modes of protein-nucleic acid binding, drive spatiotemporal organization of the nucleus and facilitate functional diversity among nuclear compartments.

## 1. Introduction

The cell nucleus is a highly organized yet dynamic organelle. It contains a variety of membrane-less compartments, including nuclear bodies and hierarchically folded chromosomes. Nuclear bodies are local concentrations of proteins and nucleic acids that are compositionally distinct from the surrounding nucleoplasm [1]. Examples include Cajal bodies, paraspeckles, nuclear speckles, histone locus bodies and the nucleolus [2]. Many of these bodies function in RNA metabolism [3,4], but some have also been implicated in DNA damage repair, transcription regulation, defense against viral infection, and genome organization [5,6,7,8].

Despite considerable cell-to-cell variability [9], advances in Chromosome Conformation Capture (3C) technology have revealed conserved features of the 3-dimensional organization of the genome [10]. Individual chromosomes occupy distinct territories within the nucleus that are further subdivided into compartments. “A” compartments are enriched for transcriptionally active genes, while “B” compartments contain silenced genes [11]. Compartments contain topologically associated domains (TADs) and chromatin loops that bring distant genomic loci together in space [10,12,13]. This hierarchical organization is thought to contribute to the spatiotemporal control of gene expression and the epigenetic status of a cell [14]. However, recent work raises questions about the generality of this structure-function relationship, as large-scale rearrangements on balancer chromosomes have little effect on gene expression patterns [15].

**Figure 1:**
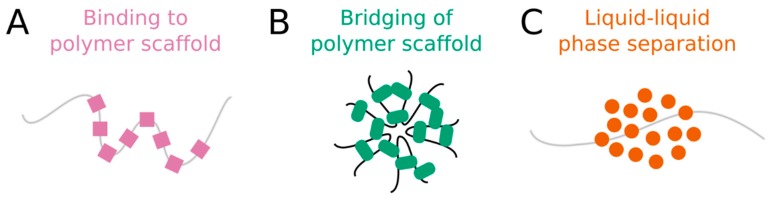
Membraneless compartments can form through at least three distinct mechanisms: (**A**) binding, (**B**) bridging, or (**C**) liquid-liquid phase separation.

Several possible mechanisms have been proposed to explain nuclear organization, specifically the formation of nuclear bodies and chromatin compartments [16]. We describe three models here, though this list is not exhaustive. First, membraneless compartments can arise through direct binding of proteins to specific sites on a nucleic acid. Cooperative binding and/or clustered binding sites yield a local enrichment of proteins that delineates a compartment along the polymer scaffold (Figure 1A). Second, the bridging of polymer segments by protein-nucleic acid interactions can induce polymer-polymer phase separation (PPPS). In this model, proteins bind to more than one nucleic acid site at a time, forming cross-links or “bridges” between binding sites. If the density of cross-links becomes sufficiently high, then the nucleic acid transitions from an extended coil to a collapsed globule [16]. The condensed nucleic acid and its protein cross-links constitute a discrete compartment (Figure 1B). Third, membraneless compartments can assemble through liquid-liquid phase separation (LLPS). This third mechanism has received so much attention in recent years that it has almost become the default explanation for nuclear compartmentalization. In living cells, LLPS is the process by which macromolecules separate, or demix, from the surrounding nucleoplasm (or cytoplasm) to form distinct, coexisting liquid phases that have different molecular compositions and material properties [17] (Figure 1C). LLPS is generally attributed to the synergy of weak multivalent interactions among intrinsically disordered regions (IDRs) of proteins and nucleic acids [1,18], which are enriched in nuclear bodies [19]. Indeed, many nuclear bodies are thought to form through LLPS in response to both physiological and pathological cues [1,20,21]. Accumulating evidence suggests that the spatial organization of chromosomes may also be attributed to LLPS [22,23]. Furthermore, LLPS has recently been proposed to explain the formation of transcriptional “condensates”, in which the transcriptional machinery concentrates on active genes [24,25,26,27].

**Figure 2:**
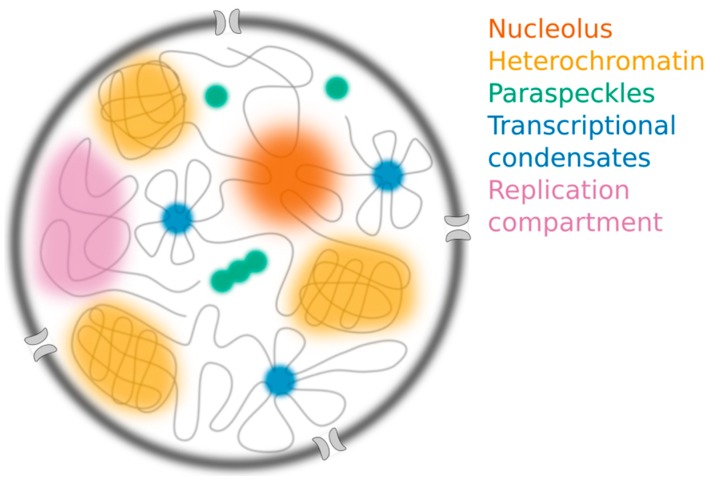
The nucleus contains many different membraneless structures, including the nucleolus (orange), constitutive heterochromatin compartments (yellow), paraspeckles (green) and transcriptional condensates (blue), which have all been proposed to assemble through liquid-liquid phase separation (LLPS). Replication compartments (purple) form following infection by herpes simplex virus.

Despite the seeming ubiquity of LLPS, the extent to which this process governs nuclear organization remains unclear for a number of reasons. First, the terminology used to describe nuclear compartments has not yet been standardized. They are often referred to as “condensates”, “droplets”, “granules”, “clusters”, “hubs”, “bodies”, “puncta”, “foci”, “complexes” or “assemblies”. This long list of terms generates unnecessary confusion and makes it difficult to compare different nuclear structures [25,27,28,29,30]. For consistency, we will follow recent precedent [30,31] and refer to compartments that form through LLPS as “condensates.” Second, the field’s efforts to establish criteria that can be used to define and identify LLPS are still evolving [5,32]. Therefore, some compartments which were confirmed to assemble by LLPS under an earlier set of criteria may no longer hold up to more rigorous tests (see Section 2.3, Section 2.4 and Section 2.5 for examples). Finally, recent theoretical work has proposed additional mechanisms that may contribute to nuclear organization, and improved experimental methods have raised doubts about the mechanism underlying certain nuclear compartments [16,33,34]. Here, we evaluate the evidence for five nuclear compartments (Figure 2) and discuss experimental strategies to rigorously distinguish LLPS from alternative processes that spatiotemporally organize the nucleus.

## 2. Distinguishing the Assembly Mechanisms Underlying Nuclear Compartments

### 2.1. The Nucleolus

The nucleolus has played a critical role in defining and understanding LLPS in the nucleus. As early as 1946, Ehrenberg described the nucleolus as a coacervate, or “a separated phase out of a saturated solution”. He showed that the nucleolus is sensitive to changes in temperature; scales with the size of the nucleus; and adopts a spherical shape [35]. In 2011, Brangwynne and colleagues further characterized the liquid-like nature of the nucleolus, using fusion kinetics to estimate its viscosity [36]. Thereafter, due to its large size (microns) and temporal stability (minutes to hours), the nucleolus has become the quintessential condensate for studying LLPS [37,38,39,40,41]. It now serves as a positive control for investigating the assembly mechanism of other nuclear compartments and for validating new methods [22].

In addition to defining the structural features of condensates (i.e., shape [36], size [40] and subcompartmentalization [41]), the nucleolus is poised to reveal new insights into condensate function. For example, stiffening of the granular component (GC), the outer layer of the nucleolus, by light-induced protein oligomerization alters ribosomal RNA (rRNA) processing [42]. Moreover, the GC reversibly stores misfolded proteins during cell stress [43]. While this protein quality control activity is effective under transient stress conditions, the nucleolus undergoes a liquid-to-solid transition during prolonged stress resulting in irreversible aggregation. These recent studies provide further support for a functional relationship between the material properties of a condensate and its cellular activity [1]. Finally, since the nucleolus occupies a large portion of the nuclear volume, it may play a more central role in nuclear organization. Indeed, a new genome-wide mapping technique showed that the nucleolus mediates interactions between multiple chromosomes simultaneously, bringing inactive regions together in space [44].

Early observations of the nucleolus and cytoplasmic P granules [36,45] led Hyman and colleagues to propose a set of criteria for defining LLPS in cells [32]. Specifically, they posited that condensates will:

1. Maintain a spherical shape;
2. Fuse after touching; and
3. Contain mobile molecules that undergo internal rearrangement and external exchange.

The former two criteria were observed for nucleoli in plant root meristems [35] and *Xenopus laevis* oocytes using transmitted light microscopy [36]. The latter was first demonstrated in mammalian cells [46], and later in *Xenopus* oocytes [41], by fluorescence recovery after photobleaching (FRAP) in which nucleolar components rearranged and exchanged within ~30–60 s. Although additional metrics for LLPS have been described (for example, deformation in response to applied force [36]; concentration-dependent size scaling [40]; or sensitivity to 1,6-hexanediol, which is widely used but not definitive [47]), the above criteria are considered the “gold standard” for demonstrating that a cellular compartment forms through LLPS (Table 1).

### 2.2. Constitutive Heterochromatin Compartments

Studies of chromatin organization have traditionally focused on DNA-binding proteins, like the cohesin complex [51] and the post-translational modification of histones [52]. Yet recent evidence suggests that these factors may act through LLPS to drive formation of constitutive heterochromatin domains. Purified human heterochromatin protein 1α (HP1α) undergoes LLPS in a phosphorylation-dependent manner in vitro [53]. Moreover, its *Drosophila* homolog, HP1a, demixes from the nucleoplasm to form spherical condensates that fuse, round up and fully recover in early embryos [22]. Nevertheless, HP1a also displays some behaviors that are not consistent with LLPS. For example, HP1a compartments appear nearly circular (in 2D) in early nuclear cycles but become progressively less circular in later cycles. FRAP measurements indicate that an increasing fraction of HP1a molecules becomes immobile over this time. In addition, HP1α compartments in mammalian tissue culture cells are not fully dispersed by 1,6-hexanediol [27,47,54]. Together, these observations suggest that heterochromatin compartments initially form through LLPS but that additional processes occur and become more dominant over time [22,53].

The progressive loss of liquid-like behavior of HP1a compartments during *Drosophila* embryogenesis [22] may be due to binding and/or bridging within the compartment. For example, concentration of DNA-binding proteins within liquid-like condensates could lead to strong cooperative binding or formation of bridging interactions that immobilize proteins and preclude the rearrangement of molecules necessary for maintaining a spherical shape. Intriguingly, HP1a variants with a point mutation in either the dimerization domain or the non-histone protein-binding domain have a significantly smaller immobile fraction than the wild-type protein in S2 cells [22]. These data suggest that bridging plays an important role in the maturation of heterochromatin compartments. Still, fluorescence correlation spectroscopy (FCS) showed that HP1a diffuses slowly and in a coordinated manner across the heterochromatin-euchromatin border in mammalian cells, indicating the presence of a phase boundary [22] (Table 1). Together, the mutant phenotypes and FCS results suggest that both bridging and LLPS contribute to the formation of heterochromatin compartments.

### 2.3. Paraspeckles

Paraspeckles are nuclear bodies that assemble around a long noncoding RNA (lncRNA) in mammalian cells. The long isoform of nuclear paraspeckle assembly transcript 1 (NEAT1_2) serves as a scaffold for RNA-binding proteins and is a critical structural component of paraspeckles [8]. LLPS has also been put forward as a mechanism contributing to paraspeckle assembly [55,56]. However, several lines of evidence raise doubts about this conclusion and instead support the bridging model [16].

First, the formation and maintenance of paraspeckles depend on a polymer scaffold. Paraspeckles assemble near endogenous or inducible sites of NEAT1 transcription and disassemble when NEAT1 is knocked down or repressed [57]. Yet active transcription is not strictly required, as *lacO*/LacI tethering of NEAT1 RNA leads to de novo assembly of paraspeckles [58]. Importantly, tethering of the core paraspeckle proteins PSPC1, NONO or SFPQ fails to support paraspeckle assembly [48]. LLPS often occurs on nucleic acid scaffolds, but condensates can exist independently of the polymer. For example, rRNA stabilizes the nucleolus and accelerates its coarsening kinetics, but extranucleolar droplets still form in vitro and in vivo in the absence of rRNA [39] or rDNA [59]. In contrast, compartments formed through PPPS cannot exist without a polymer scaffold [16]. Consistent with this prediction from the bridging model, paraspeckles are not detected in NEAT1^−/−^ cells unless NEAT1_2 is supplied on a rescue plasmid [60]. The requirement of NEAT1 RNA for paraspeckle assembly suggests that LLPS is not the primary mechanism responsible for assembling this compartment.

Second, the shape and size of paraspeckles are not characteristic of liquid-like condensates. LLPS generates spherical droplets that fuse upon touching [32]. Individual paraspeckles are approximately spherical, with a well-defined diameter of ~350 nm [49,50]. However, when NEAT1 expression increases, paraspeckles maintain a constant width and elongate along one axis rather than growing isotropically in all directions [49,50]. Super-resolution imaging revealed that these elongated structures are linear chains of paraspeckles, indicating that these compartments neither fuse nor relax into larger spheres [61]. Moreover, condensates do not have a characteristic size. Instead, their size distribution is determined by component concentration and fusion dynamics, as observed for the nucleolus [36,40]. Yet the size of paraspeckles seems to be fixed by the NEAT1 scaffold, not the concentration of self-interacting proteins. For example, overexpression of NEAT1, but not PSPC1, increases paraspeckle number [57]. Paraspeckle size (through clustering/elongation) increases following inhibition of the proteasome or stimulation of the immune response, perturbations which upregulate NEAT1 transcription without affecting paraspeckle protein levels [7,50,57]. Finally, paraspeckles assembled on a truncated “mini” version of NEAT1 (~10 kb compared to the native 23 kb in humans) are small, with a diameter of only 200 nm [56]. Together, these observations demonstrate that the size and shape of paraspeckles are determined strictly by the length and abundance of NEAT1, contradicting two criteria for LLPS (Table 1).

Third, paraspeckles do not appear to buffer the concentration of their protein components. Condensates are in equilibrium with a dilute phase whose concentration is equal to the saturation concentration (c_sat_) of the phase-separating components. For instance, nucleoli buffer the concentration of nucleolar components in the nucleoplasm, which remains constant at c_sat_ [40]. However, when paraspeckles elongate following treatment with the proteasome inhibitor MG132, NONO and SFPQ are depleted from the nucleoplasm by ~50% [50]. Since these proteins do not maintain a stable (saturated) concentration in the nucleoplasm, their accumulation in paraspeckles cannot be governed by LLPS.

**Figure 3:**
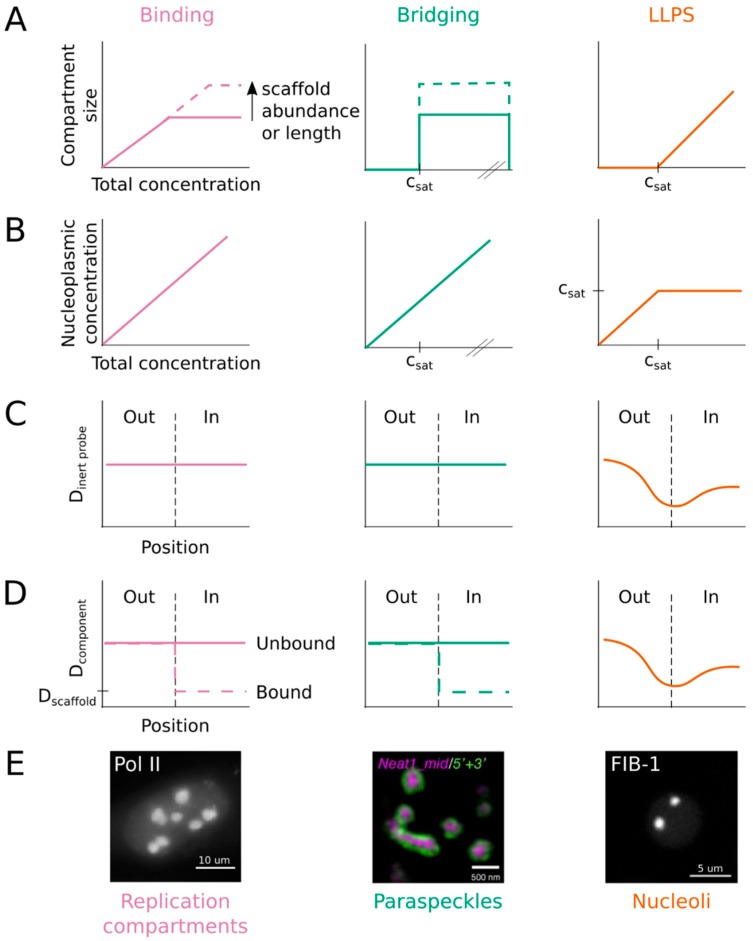
Concentration dependence and diffusion across boundary represent useful criteria for distinguishing among various mechanisms for nuclear compartmentalization. (**A**) Each model predicts a distinct relationship between compartment size and component concentration. (**B**) LLPS can buffer the nucleoplasmic concentration, while binding and bridging mechanisms cannot. (**C**) Inert probes freely diffuse through compartments formed by binding or bridging, but their mobility is hindered by the phase boundary. (**D**) Component molecules move similarly to inert probes except when bound to the polymer scaffold. (**E**) Despite their spherical shape and molecular dynamics, replication compartments and paraspeckles are not consistent with LLPS. Images are reprinted from refs. [33,40,61] under the Creative Commons license: http://creativecommons.org/licenses/by/4.0/.

Paraspeckles share many properties with cellular condensates (Table 1). Their core proteins (but notably not NEAT1) are mobile and rapidly exchange with the surrounding nucleoplasm [48]. They are enriched for proteins containing prion-like domains, a type of IDR [55]. They have a core-shell architecture [61], similar to the nucleolus [41] and stress granules [62]. They are sensitive to 1,6-hexanediol [56]. Nevertheless, none of these properties are definitive for LLPS [5,32]. Therefore, given the necessity of a NEAT1 scaffold, their elongated shape and fixed size, and their ability to deplete proteins from the nucleoplasm, we propose that paraspeckles form through bridging rather than LLPS. To test this hypothesis, it will be important to repeat the proteasome inhibition assay for other paraspeckle proteins, especially FUS and RBM14, which form condensed phases in vitro [55,63] and are essential for paraspeckle assembly in vivo [60]. Such an experiment would indicate whether FUS and RBM14 undergo LLPS at paraspeckles in vivo or whether they contribute to paraspeckle assembly through an alternative mechanism. More generally, we urge the field to adopt more stringent criteria for classifying compartments as condensates formed by LLPS. One such metric could be the response to changes in component concentration. Unlike spherical shape or molecular mobility, alternative models for compartment assembly do not predict a saturation threshold and/or compartment size scaling [5,32], making this experiment a useful diagnostic (Figure 3).

### 2.4. Transcriptional “Condensates”

Transcription brings together various cis-regulatory elements (e.g., promoters, enhancers) and trans-acting factors (e.g., transcription factors (TFs), co-activators) in close proximity at active genes [64]. This highly coordinated process is rapid and specific, facilitating the precise control of gene expression. In addition, the transcriptional machinery is comprised of proteins with IDRs and multivalent interaction domains that are subject to reversible posttranslational modification [65]. Inspired by these similarities with membraneless organelles [66], LLPS was recently proposed to explain the formation and regulation of super-enhancers, genomic regions that contain clusters of multiple enhancers which can activate transcription of multiple genes simultaneously [24,67].

Consistent with this hypothesis, many TFs form concentrated dynamic compartments that interact with RNA polymerase II (Pol II) [24,67]. For example, the IDRs of three RNA-binding proteins—FUS, EWS and TAF15, collectively known as “FET” proteins—undergo phase separation in vitro to form hydrogels [68] or liquid condensates [69] that bind the intrinsically disordered C-terminal domain (CTD) of Pol II. When fused to the GAL4 DNA-binding domain, these IDRs activate transcription of a luciferase reporter, suggesting that these interactions are functional in vivo [68]. Moreover, the FET IDRs recruit Pol II and exhibit liquid-like properties in live cells. They form large spherical compartments that are visible by bright-field microscopy, reflecting a change in refractive index that may arise from a distinct phase separated from the surrounding nucleoplasm [27]. The molecules within these compartments are mobile, with FRAP times on the order of minutes, and disperse upon treatment with 1,6-hexanediol. Importantly, the number and size of compartments increase above a minimum protein concentration [27]. Thus, as expected for LLPS, the FET IDRs buffer their nucleoplasmic concentration by forming condensates whose size scales with concentration above a saturation threshold, c_sat_. Furthermore, super-resolution imaging of these TFs [27], as well as the co-activators BRD4 [25] and Mediator [29], suggests that they form compartments that recruit Pol II even at endogenous expression levels.

TFs and co-activators associate with Pol II at promoters to initiate transcription. However, Pol II must coordinate its activity with different sets of trans-acting factors during elongation and termination [64]. New studies suggest that LLPS may not only facilitate transcription initiation, but also elongation and, possibly indirectly, termination. For example, the regulatory subunit of positive transcription elongation factor b (P-TEFb), cyclin T1, which contains a histidine-rich IDR, forms condensates in vitro that recruit Pol II CTD [70]. The cyclin T1 IDR is required for stimulating hyperphosphorylation of the CTD by CDK9, the catalytic subunit of P-TEFb. While phosphorylation prevents the CTD from partitioning into TAF15 hydrogels [68], it enhances condensation of cyclin T1 [70]. In cells, cyclin T1 concentrates at active transcription sites to form compartments that fuse, exchange molecules with the nucleoplasm, and are sensitive to 1,6-hexanediol. Similar observations were made for the splicing factor SRSF2, which occasionally colocalizes with Pol II [26]. In vitro, phosphorylation of the CTD increases its incorporation into SRSF2 condensates, while decreasing its affinity for Mediator condensates. Even alone, the CTD phase separates in a phosphorylation-dependent manner [28]. Although more evidence is needed to confirm LLPS as the underlying mechanism in vivo, transcription appears to be controlled by several different compartments that concentrate Pol II with the appropriate accessory factors at successive locations along an active gene.

Together, these results support a new model for transcriptional regulation and efficiency [64]. At initiation, the CTD is unphosphorylated and associates with compartments formed by TFs, Mediator and BRD4 [25,27,29,68]. Phosphorylation of the CTD causes Pol II to dissociate from these “transcription initiation compartments” at the promoter and instead enter “transcription elongation compartments” formed by P-TEFb [70] and/or various splicing factors, including SRSF2 [26], throughout the open reading frame. Finally, dephosphorylation of the CTD by protein phosphatase 1 may induce compartment disassembly and release Pol II for transcription termination [71,72].

### 2.5. Viral Replication Compartments

Pol II forms dozens of small (~100 nm), transient (~10 s) compartments, and several large (>300 nm), stable (>100 s) ones, throughout the nucleus of healthy mammalian cells [29,73]. Following infection by herpes simplex virus type 1 (HSV1), Pol II massively redistributes and concentrates within replication compartments (RCs), which hijack host factors to replicate and transcribe the viral genome [33]. Like transcriptional “condensates” at super enhancers [25,27,29], RCs meet the three basic criteria for LLPS (Table 1). They adopt a spherical shape; they undergo fusion; and their molecular components are mobile, though FRAP slows over the course of infection [33]. Nevertheless, closer examination suggests that RCs instead form through direct binding to DNA rather than LLPS (Figure 3). For example, unlike transcriptional “condensates” [28], RCs are not affected by truncation or extension of the CTD and are stable in the presence of 1,6-hexanediol [33]. These observations suggest that weak multivalent interactions between IDRs are not the dominant force driving RC formation. Furthermore, the area of RCs grows over the course of infection as the copy number of the HSV1 genome increases [33]. When viral DNA replication is inhibited, RCs remain small even six hours post infection. Thus, as for paraspeckles, the size of RCs scales with the abundance of a polymer scaffold rather than protein concentration. Finally, and most tellingly, Pol II diffuses freely across the supposed border separating RCs from the nucleoplasm [33]. Single-molecule tracking (SMT) showed that Pol II molecules move with the same apparent diffusion coefficient inside RCs as they do outside, or in uninfected cells. Moreover, their motion was not restricted when entering or exiting RCs. Fluorescence loss in photobleaching measurements confirmed that Pol II exchanges between RCs and the nucleoplasm at similar rates throughout infection and as fast as in uninfected nuclei. While the diffusion of Pol II does not change during infection, the fraction of “immobile”, or DNA-bound, molecules increases dramatically inside RCs of infected cells. In other words, RCs appear to form by binding of Pol II (and other host and viral factors [74]) to the HSV1 genome. Importantly, these results contradict expectations for LLPS. A phase boundary imposes a barrier to diffusion, as observed for nucleoli and heterochromatin compartments using fluorescence correlation spectroscopy [22]. Yet SMT analysis of RCs detected no evidence of a phase boundary [33].

This example serves as a cautionary tale and underscores the inadequacy of the current consensus criteria for LLPS. RCs exhibit several properties that are consistent with LLPS (Table 1) but ultimately this conclusion proved incorrect. Indeed, SMT provides complementary information that can be used to discriminate between LLPS and alternate models (Figure 3).

### 2.6. Other Nuclear Compartments

In addition to the compartments discussed here, LLPS has been invoked in the formation of many other nuclear structures: Cajal bodies, PML bodies, nuclear speckles, and histone locus bodies [75,76,77,78]; facultative heterochromatin [79,80] and the inactivated X chromosome [81]; the central channel of the nuclear pore complex [82]; the mitotic spindle [83]; and the centromere [84]. Nevertheless, given the unexpected behavior observed for constitutive heterochromatin compartments, paraspeckles and RCs, we caution the field against jumping to the conclusion that LLPS governs all membraneless nuclear compartments and their cytoplasmic counterparts.

## 3. Evolving Metrics for LLPS

Here, we elaborate on two experimental strategies which can be used to test the LLPS hypothesis further than conventional fusion or FRAP assays [5,32] (Figure 3).

### 3.1. Concentration Dependence

Condensates are defined by a saturation threshold, c_sat_. When the concentration of a molecular component is below c_sat_, it remains dissolved in the nucleoplasm and the compartment does not form. When it exceeds c_sat_, then the components condense into a liquid phase. The volume of the condensed phase scales with the degree of supersaturation, such that the nucleoplasm is buffered and maintains a concentration equal to c_sat_ (Figure 3A,B, right column). In contrast, the size of compartments assembled by binding or bridging do not scale with component concentration, but rather with the length or abundance of a polymer scaffold (Figure 3A). Moreover, they do not buffer the nucleoplasm (Figure 3B).

These properties can be evaluated experimentally by manipulating either the concentration of components (i.e., through inducible expression or degradation) or the size of the nucleus (i.e., through growth and development or genetic perturbation). Indeed, this strategy was applied successfully to endogenous nucleoli in dividing embryos and developing larvae of *C. elegans* [38,40]. It has also been used for artificial nuclear bodies formed by the FET IDRs [5,22,27] and the Nephrin intracellular domain [85]. In each of these cases, the condensate only formed when the component concentration exceeded a threshold (c_sat_), above which its size scaled with total concentration and the nucleoplasmic concentration was buffered around c_sat_. Though not measured directly, multiple studies indicate that paraspeckles do not exhibit these properties [49,50,56,57], contradicting claims that they assemble through LLPS [55,56] (Figure 3E). We recommend that concentration dependence, specifically that of compartment size and nucleoplasmic concentration, be added to the list of criteria necessary to define LLPS (Table 1, Figure 3).

### 3.2. Diffusion Across Boundary

Condensate components and inert probes alike must break cohesive interactions to cross the interface between a condensate and the surrounding nucleoplasm. This is energetically unfavorable, so molecules tend to move away from the interface, or more slowly across it. Thus, the presence of a phase boundary restricts the motion of molecules across the interface, reducing the diffusion rate at the boundary (Figure 3C,D). In contrast, there is no interface to impede diffusion into or out of a compartment formed by binding or bridging. Inert probes move through these compartments with no change in diffusion rate (Figure 3C). Similarly, the diffusion of condensate components is not affected, unless they directly bind to or bridge the polymer scaffold (Figure 3D).

Diffusion across a compartment’s boundary can be determined using FCS or SMT. For example, FCS measurements demonstrated that the conserved nucleolar protein fibrillarin diffuses more slowly at the nucleolus-nucleoplasm boundary then it does on either side of the boundary [22], consistent with LLPS (Figure 3E). *Drosophila* HP1a and mammalian HP1α, as well as an inert YFP construct, show similar behavior near the heterochromatin-euchromatin boundary, indicating the presence of a phase boundary around constitutive heterochromatin compartments. Unexpectedly, SMT revealed that Pol II diffusion does not change when entering or exiting RCs. This result is strong evidence in support of binding (or bridging) over LLPS as the mechanism driving RC assembly (Figure 3E). In addition to average diffusion rates, SMT provides detailed accounting of sub-populations of molecules with different mobility states. This information is necessary to interpret bulk FRAP measurements more accurately [33]. Furthermore, we anticipate that SMT will become an increasingly important technique for characterizing nuclear compartments as more structures are identified using super-resolution microscopy [29]. Indeed, spherical shape and molecular mobility are difficult to assess for compartments that fall below the diffraction limit and SMT offers a powerful approach for examining such structures. Thus, diffusion across boundaries is another criterion that should be used to define LLPS (Table 1, Figure 3).

## 4. Conclusions

The discovery of LLPS has fundamentally changed our view of nuclear organization. Further elucidation of the biophysical processes governing nuclear compartments promises to uncover a deeper understanding of not only the dynamic structure of the nucleus, but also its numerous functions. The three models discussed above—binding, bridging and LLPS—likely represent just a subset of potential mechanisms operating in the nucleus, yet they give rise to distinct properties that could have profound effects on compartment function. For example, one possible function of LLPS that is often cited is concentration buffering [5], which may reduce noise in gene expression [86]. Since binding and bridging cannot regulate the concentration of components in the nucleoplasm (Figure 3B), this activity is not possible for non-LLPS compartments such as RCs and paraspeckles. Furthermore, LLPS creates a phase boundary that restricts the diffusion of components and inert molecules alike (Figure 3C, D). This property makes condensates selectively permeable and capable of maintaining a biomolecular composition that is different from the nucleoplasm [5], which could either accelerate or inhibit biochemical reactions. In contrast, the lack of a boundary for compartments formed through binding and bridging allows free access to the interior (Figure 3C,D). Although the binding or bridging component is locally concentrated on the polymer scaffold, other molecules are neither sequestered nor excluded.

Importantly, these models are not mutually exclusive. Indeed, the conversion from one mechanism to another, or the simultaneous combination of two or more, may allow cells to adapt the function of their nuclear compartments to environmental and developmental changes. For example, constitutive heterochromatin compartments appear to form as LLPS-mediated condensates that eventually incorporate bridging interactions later in development [22]. Different processes may also contribute to other chromatin domains; bridging may dominate at lower-length scales to form loops and TADs [12,87], while LLPS appears to mediate higher-order compartments [16,44,51]. Post-translational modifications of proteins and nucleic acids may facilitate the transition among different mechanisms by altering the affinity and/or valency of intermolecular interactions. Rapid development of new optogenetic and gene-editing techniques provide tools that can used to assess the functional relevance of these hypotheses [88,89,90]. These experimental methods, combined with an expanded set of criteria that can accurately identify the mechanisms underlying nuclear organization, will propel the field forward, revealing deeper insights into the varied functions of nuclear compartments.

## Figures and Tables

**Table 1:** Additional criteria are necessary for identifying liquid-liquid phase separation (LLPS).

LLPS Criterion	Nucleolus	Heterochromatin	Paraspeckles	Transcriptional “Condensates”	Replication Compartments
Spherical shape	Yes	**Yes and no**	**Yes and no**	Yes	Yes
Fusion	Yes	Yes	**No**	Yes	Yes
Molecular mobility ^1^	Yes	**Yes and no**	Yes	Yes	Yes
Concentration dependence	Threshold, size scaling	ND ^3^	**No c_sat_; size scaling with scaffold length/abundance, not [protein]**	Threshold, size scaling	**Size scaling with DNA scaffold**
Diffusion across boundary ^2^	Increased variance; decreased rate	Increased variance; decreased rate	ND ^3^	ND ^3^	**Unrestricted diffusion**
References	[22,36,40,46]	[22]	[48,49,50]	[25,27,29]	[33]

^1^ Molecular mobility is traditionally measured by fluorescence recovering after photobleaching (FRAP). ^2^ Diffusion across boundary can be measured by fluorescence correlation spectroscopy (FCS) or single-molecule tracking (SMT). ^3^ ND, not determined. Observations in bold are inconsistent with LLPS. Additional criteria proposed in Section 3 are shaded in gray.
